# Cisplatin-induced activation of TGF-β signaling contributes to drug resistance

**DOI:** 10.32604/or.2023.030190

**Published:** 2023-11-15

**Authors:** SAYAKA IMATSUJI, YUKIKO UJIE, HIROYUKI ODAKE, MASAYA IMOTO, SUSUMU ITOH, ETSU TASHIRO

**Affiliations:** 1 Department of Biosciences and Informatics, Faculty of Science and Technology, Keio University, Yokohama, 223-8522, Japan; 2 Department of Neurology, Juntendo University Graduate School of Medicine, Tokyo, 113-8421, Japan; 3 Laboratory of Biochemistry, Showa Pharmaceutical University, Tokyo, 194-8543, Japan

**Keywords:** Cisplatin, EMT, Chemo-resistance, TGF-β

## Abstract

Growing evidence suggests an association between epithelial-mesenchymal transition (EMT), a hallmark of tumor malignancy, and chemoresistance to a number of anti-cancer drugs. However, the mechanism of EMT induction in the process of acquiring anti-cancer drug resistance remains unclear. To address this issue, we obtained a number of cisplatin-resistant clones from LoVo cells and found that almost all of them lost cell-cell contacts. In these clones, the epithelial marker E-cadherin was downregulated, whereas the mesenchymal marker N-cadherin was upregulated. Moreover, the expression of EMT-related transcription factors, including Slug, was elevated. On the other hand, the upregulation of other mesenchymal marker Vimentin was weak, suggesting that the mesenchymal-like phenotypic changes occurred in these cisplatin-resistant clones. These mesenchymal-like features of cisplatin-resistant clones were partially reversed to parental epithelial-like features by treatment with transforming growth factor-β (TGF-β) receptor kinase inhibitors, indicating that TGF-β signaling is involved in cisplatin-induced the mesenchymal-like phenotypic changes. Moreover, cisplatin was observed to enhance the secretion of TGF-β into the culture media without influencing TGF-β gene transcription. These results suggest that cisplatin may induce the mesenchymal-like phenotypic changes by enhancing TGF-β secretion, ultimately resulting in drug resistance.

## Introduction

Cancer is a leading cause of death worldwide. In 2019, more than 10 million people died of cancer [1]. Although numerous different methods of cancer therapy have been developed, including surgery, radiation therapy, immunotherapy, and endocrine therapy, chemotherapy remains the most common. Currently, a number of anti-cancer drugs with different modes of action are commercially available. Conversely, it is well established that prolonged treatment with anti-cancer drugs causes cancer cells to become drug-resistant. Drug resistance can be associated with a variety of mechanisms, including enhanced efflux of drugs, genetic factors (gene mutations, amplifications, and epigenetic alterations), growth factors, increased DNA repair capacity, and elevated metabolism of xenobiotics [2,3]. Recent studies have suggested that epithelial-mesenchymal transition (EMT) is a major contributor to anti-cancer drug resistance. For example, breast cancer cells that undergo EMT are more resistant to existing anti-cancer drugs than are cells that do not undergo this process [4]. Most lung cancer cells that are resistant to epidermal growth factor receptor (EGFR) inhibitor erlotinib exhibit a mesenchymal phenotype [5]. The general mechanism regarding EMT-associated drug resistance is accompanied by increased drug efflux, slow cell proliferation and avoiding apoptosis signaling pathway. In addition, escaping from the immune response is also considered to be another important mechanism by which EMT contributes to drug resistance [6].

On the other hand, in the process of acquiring drug resistance, EMT was reported to occur. For example, continuous exposure to paclitaxel was reported to induce EMT in epithelial ovarian cancer cells [7]. However, it is poorly understood the molecular mechanism of EMT-associated drug resistance as well as EMT induction in the process of acquiring drug resistance.

EMT is recognized as a highly conserved cellular program. During EMT, tight cell-cell junctions among polarized epithelial cells are destroyed. The epithelial cells then disperse and differentiate into nonpolarized, motile, and invasive mesenchymal cells. Thus, the expression of a number of molecules that are associated with cell-cell adhesion, polarity, and the extracellular matrix (ECM) is largely altered. For example, the expressions of the epithelial cell-cell adhesion molecule E-cadherin and the mesenchymal cell-cell adhesion molecule N-cadherin is decreased and enhanced, respectively [8]. These processes are mediated by gene activation of transcription factor(s) that is termed EMT-TF(s), and these factors include Snail, Slug, Zeb, and Twist. The activation of EMT-TFs is initiated by extrinsic signals (e.g., growth factor signaling). TGF-β [9], hepatocyte growth factor (HGF) [10], EGF [11], insulin-like growth factor (IGF) [12], and fibroblast growth factor (FGF) [13,14] have been reported to induce EMT. Among these, TGF-β is the most well-known EMT-inducing factor. In mammals, there are three genetically distinct TGF-β ligands (TGF-β1-3). TGF-β signaling is initiated by the binding of TGF-β to the widely expressed TGF-β type II receptor (TβRII). Activated TβRII then recruits the TGF-β type I receptor (TβRI) to phosphorylate serine and threonine residues in the glycine-serine repeat (GS) domain located in the juxtamembrane region. Consequently, TβRI kinase becomes active, and the TβRI kinase phosphorylates the two extreme C-terminal serine residues of the receptor-regulated Smads (R-Smads that include Smad2 and Smad3). Once phosphorylated, the two R-Smads form heteromeric complexes with Smad4, and these complexes subsequently translocate to the nucleus and transcriptionally regulate several genes (including EMT-TFs) through interactions with a variety of transcriptional activators or repressors [15].

In this study, to address the mechanism of EMT induction in the process of acquiring anti-cancer drug resistance, we first examined which combination of cell lines and anti-cancer drugs could induce drug resistance. As a result, we established cisplatin-resistant LoVo cells (cisplatin^r^/LoVo cells) through continuous exposure of these cells to cisplatin. While LoVo cells exhibited an epithelial phenotype, all these cisplatin^r^/LoVo cells possessed a mesenchymal-like phenotype which are consistent with the previous reports. Therefore, we tried to shed light on the mechanism by which the mesenchymal-like phenotypic changes occurred in the process of acquiring cisplatin-resistance. As a result, we indicated that cisplatin enhanced the secretion of TGF-β1 which might result in the mesenchymal-like phenotypic changes and subsequent acquisition of cisplatin-resistance.

## Materials and Methods

### Materials

These reagents were purchased from the indicated sources, including cisplatin (FUJIFILM Wako Pure Chemical #039-20093, Tokyo, Japan), TGF-β1 (R&D Systems #240-B, Minneapolis, MN), SB431542 (Sigma-Aldrich #S4317, St. Louis, MO), and TβRI kinase inhibitor II (TK-II, RepSox) (Merck Millipore #616452, Billerica, MA). The following antibodies were obtained from the indicated sources, including anti-E-cadherin (#610181), anti-N-cadherin (#610920), and anti-fibronectin (#610077) that were purchased from BD Biosciences (San Jose, CA). The anti-vimentin (#V6389) antibody was purchased from Sigma-Aldrich. Anti-slug (#9585) antibody was obtained from Cell Signaling Technology (Danvers, MA, USA). Anti-phospho-Smad3C (#28031) antibody was purchased from IBL (Gumma, Japan). Furthermore, anti-Smad2/3 (sc-8332) and anti-β-actin (sc-69879) antibodies were purchased from Santa Cruz Biotechnology (Santa Cruz, CA).

### Cell culture

Human colorectal adenocarcinoma-derived cell line LoVo, of which some loci were different from those of an original LoVo (ATCC: CCL-29), was kindly provided by Kirin corporation. Human colorectal adenocarcinoma-derived cell line HCT116, HT29, and DLD-1, human non-small cell lung cancer (NSCLC) adenocarcinoma-derived cell line A549, and human prostate cancer-derived cell line LNCaP were obtained from the American Type Culture Collection (ATCC, Rockville, MD, USA). LoVo, HCT116, HT29, DLD-1, and LNCaP cells were maintained in Roswell Park Memorial Institute (RPMI) medium 1640 (RPMI1640; Nissui Pharmaceutical, Tokyo, Japan) containing 10% fetal bovine serum (FBS; JRH Biosciences), 100 U/mL of penicillin G (Sigma #13752), and 0.1 mg/mL of kanamycin (Sigma #K1377). A549 cells were cultured in Dulbecco’s modified Eagle’s medium (DMEM; Nissui Pharmaceutical) supplemented with 10% FBS, 100 U/mL of penicillin G, and 0.1 mg/mL of kanamycin. All cell lines were maintained at 37°C in a 5% CO_2_–95% air atmosphere. Unfortunately, we conducted a study using Mycoplasma-infected cells in some experiments.

### Western blotting

Cells were lysed with RIPA buffer (25 mM HEPES [pH 7.8], 1.5% Triton X-100, 1% sodium deoxycholate, 0.1% SDS, 0.5 M NaCl, 5 mM EDTA, 50 mM NaF, 100 mM Na_3_VO_4_, 1 mM phenylmethylsulfonyl fluoride (PMSF), and protease inhibitor cocktail (Roche #11836153001, Basel, Switzerland)). Protein lysates were separated by SDS-PAGE and transferred to PVDF membranes (Merck Millipore #IPV00010). The membranes were blocked with 5% non-fat milk in tris-buffered saline (TBS) with 0.1% Tween-20 (TBS-Tween), shaken at 24°C for 30 min, and incubated with the indicated primary antibodies (1:1000 dilution) overnight at 4°C. Then, the membranes were washed three times with TBS-Tween and incubated with the appropriate horseradish peroxidase (HRP)-conjugated secondary antibodies (Cytiva #NA934 or #NA931, Marlborough, MA) (1:2500 dilution) for 1 h at 24°C. After three washes with TBS-Tween every 10 min, chemiluminescence was detected using an Immobilon Western Kit (Merck Millipore #WBKLS0050) and ChemiDoc XRS+ (Bio-Rad, Hercules, CA, USA). At least two biological replicates were performed for each assay.

### qPCR

Briefly, total RNA was extracted using a RNeasy Mini Kit (Qiagen, Venlo, Netherlands, #74104). Total RNA (1 μg) was reverse transcribed into cDNA using MM-LV reverse transcriptase (Promega, Madison, WI, USA, #M5313) according to the manufacturer’s instructions. Quantitative PCR was performed on a Thermal Cycler Dice (TaKaRa, Shiga, Japan) using the SYBR Premix ExTag Kit (TaKaRa #RR820A). The RPL37A was used as an internal control, and the gene expression was normalized to the RPL37A to calculate the relative expression level using 2^−ΔΔCt^ method. The primers are listed in Table 1.

**Table 1:** Primers used in this study

Gene	Forward primer 5′–3′	Reverse primer 5′–3′
RPL37A	ATTGAAATCAGCCAGCACGC	GCAGGAACCACAGTGCCAGATCC
E-cadherin	TGCCCAGAAAATGAAAAAGG	GTGTATGTGGCAATGCGTTC
N-cadherin	GACAATGCCCCTCAAGTGTT	CCATTAAGCCGAGTGATGGT
Fibronectin	CCAACCTACGGATGACTCGT	GCTCATCATCTGGCCATTTT
Slug	TGATGAAGAGGAAAGACTACAG	GCTCACATATTCCTTGTCACAG
ZEB2	TTCCTGGGCTACGACCATAC	TGTGCTCCATCAAGCAATTC
Twist	ACAAGCTGAGCAAGATTCAGACC	TCCAGACCGAGAAGGCGTAG
TGF-β1	TGAACCGGCCTTTCCTGCTTCTCATG	GCGGAAGTCAATGTACAGCTGCCGC

### Detection of apoptosis by flow cytometry

To determine the percentage of apoptotic cells, propidium iodide (PI; Sigma #P4170) staining was performed as described previously [16]. Briefly, cisplatin-treated cells were harvested with trypsin and fixed with ice-cold 70% ethanol for at least 1 h at 4°C. The cells were then incubated with RNase/phosphate-buffered saline (PBS) and stained with a 50 mg/mL PI solution. 10,000 cells were counted in each assay, and PI fluorescence intensity was measured using a flow cytometer (Epics XL, Beckman Coulter, Brea, CA, USA), and the percentage of apoptotic cells was determined by quantifying the subG1 population.

### Confocal laser scanning microscopy

Cells were fixed with 3% paraformaldehyde for 15 min and then permeabilized with 0.5% Triton X-100 in PBS for 5 min. After rinsing the fixed cells with PBS three times, they were incubated with blocking buffer (1% bovine serum albumin in PBS) for 30 min. The fixed cells were then stained with an anti-E-cadherin (1:1000) antibody for 1 h and stained with an anti-mouse IgG-alexa-568-conjugated secondary antibody (1:1000) (Invitrogen #A-11004) for 1 h. Fluorescence imaging was performed using a FV1000 confocal laser scanning microscope system (Olympus, Tokyo, Japan).

### Anchorage-independent growth assay

Briefly, poly(2-hydroxyethyl methacrylate) (Poly-HEMA; Sigma P3932)-coated 12-well plate were prepared by the following protocol. 500 μL of 5 mg/mL Poly-HEMA in EtOH were added to each well of 12-well plate and coated by the incubation at 45°C overnight. Then, 1 × 10^5^ cells were seeded on a Poly-HEMA-coated 12-well plate and incubated. After 72 h, the floating cells were transferred to a 1.5 mL tube and centrifuged at 1,500 rpm for 5 min. The collected cells were suspended into 200 μL RPMI containing 10% FBS and then 100 μL Cell-Titer Glo (Promega #G7570) was added to the cell suspension. Ten minutes later, each luminescence was measured by Fluoroskan Ascent FL (Thermo Fisher Scientific, Waltham, MA, USA), and the luminescence signal was defined as the proliferation rate in the 3D culture. At the same time, 1 × 10^4^ cells were seeded on a 96-well plate. After cells were cultured for 72 h, 100 μL Cell-Titer Glo was added. Ten minutes later, each luminescence was measured by Fluoroskan Ascent FL, and the luminescence signal was defined as the proliferation rate in the 2D culture. Then, the ratios between the proliferation rates in 2D and 3D cultures were calculated.

### MTT assay

Briefly, cells were seeded at 5 × 10^3^ cells/well in 96-well plates and cultured overnight. Then the cells were treated with various concentrations of anti-cancer drugs. Three days later, the cells were treated with 0.5 mg/mL of 3-(4,5-dimethylthiazolyl)-2,5-diphenyltetrazolium bromide (MTT) (Sigma #475989) for 4 h at 37°C, and then added DMSO to dissolve formazan crystals formed during the reduction of yellow tetrazolium dye. Absorbance at 595 nm was measured by a Multiskan FC (Thermo Fisher Scientific, Waltham, MA, USA).

### Statical analyses

All data are presented as the mean ± standard deviation (SD). Statistical analyses were performed using a two-tailed non-paired Student’s *t*-test unless otherwise stated.

## Result

### Establishment of cisplatin^r^/LoVo cells by continuously exposing LoVo cells to cisplatin

**Figure 1:**
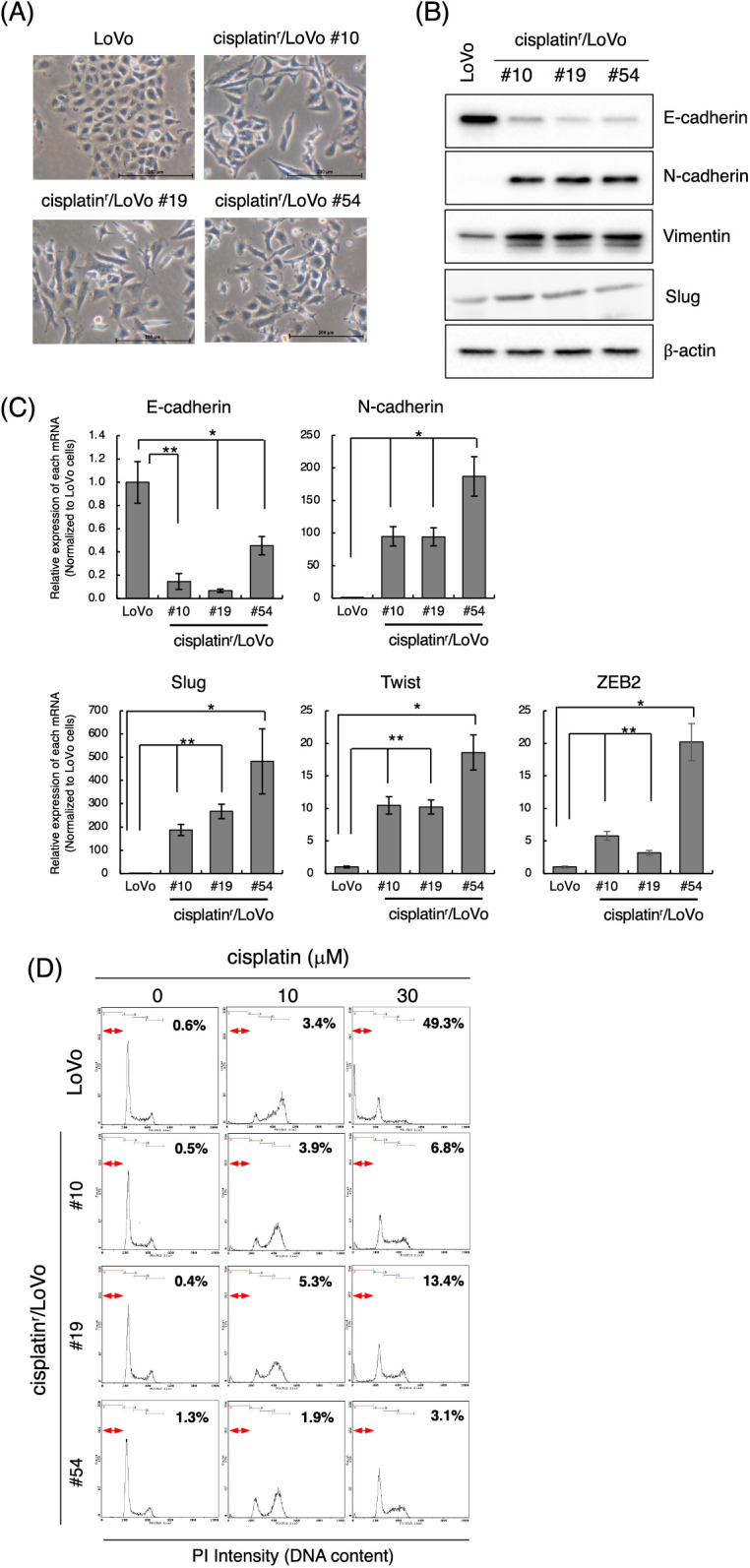
Establishment of cisplatin^r^/LoVo cells having mesenchymal-like features. (A) Morphologies of LoVo cells and cisplatin^r^/LoVo #10, #19, and #54 cells were observed under a phase-contrast microscope. Images are representative of two independent experiments. Scale bar, 200 μm. (B) LoVo and cisplatin^r^/LoVo cells were lysed, and the expression of E-cadherin, N-cadherin, Vimentin, Slug, and β-actin was detected by western blotting. Images are representative of two independent experiments. (C) Measurement of E-cadherin, N-cadherin, Slug, Twist, and ZEB2 mRNAs levels. Total RNA was extracted from exponentially growing LoVo and cisplatin^r^/LoVo #10, #19, and #54 cells. Then, the amount of E-cadherin, N-cadherin, and Slug, Twist, and ZEB2 mRNAs was evaluated with qPCR. The presented data are normalized to LoVo cells. All data are representative of three independent experiments and presented as mean SD ± (n = 3). *p < 0.05, **p < 0.01 (two-tailed Student’s test). (D) Cisplatin^r^/LoVo cells were resistant to cisplatin-induced apoptosis. LoVo cells and each cisplatin^r^/LoVo cells were treated with 10 or 30 μM of cisplatin for 48 h. Cells were fixed with 70% ethanol, stained with PI, and analyzed using flow cytometry. Percentages of the subG1 population (areas between red line) in cells treated with 30 μM cisplatin are shown. Moreover, percentages of the subG1, G1, S, and G2/M phase populations are summarized in Table 2. Images are representative of two independent experiments.

To address the mechanism of EMT induction in the process of acquiring anti-cancer drug resistance, we initially tried to establish a chemo-resistant cancer cell line. Briefly, we determined the minimum concentration of anti-cancer drugs, including cisplatin, doxorubicin, paclitaxel, and camptothecin, against several human cancer cell lines (LoVo, HCT116, MCF7, LNCaP, and PC3 cell lines) that inhibited cell growth but did not affect cell viability after 3 days of treatment evaluated by trypan blue exclusion assay (data not shown). Next, we added each anti-cancer drug at the determined concentrations and changed the culture medium containing anti-cancer drugs every 3 days. Then, the cells were continuously exposed to gradually increasing concentrations of anti-cancer drugs for 1 month. For almost all but one combination, we were unable to obtain the surviving cells (data not shown). However, after continuously exposing LoVo cells to cisplatin for over one month, we totally obtained 54 clones of cisplatin-resistant LoVo (cisplatin^r^/LoVo) cells. Whereas LoVo cells possess tight cell-to-cell contacts, the morphologies of most cisplatin^r^/LoVo cells changed to a loss of cell-to-cell contacts and an elongated shape with filamentous protrusions that is the typical morphology of mesenchymal cells (Fig. 1A). Therefore, we next examined the expression of epithelial marker E-cadherin and mesenchymal marker N-cadherin. In LoVo cells, the protein expression of E-cadherin was high, while that of N-cadherin was undetectable or low. On the hand, among 54 clones, E-cadherin downregulation and N-cadherin upregulation were observed in 42 clones. Then, we selected cisplatin^r^/LoVo #10, #19, and #54 in these 42 clones for further study because E-cadherin and N-cadherin were strongly downregulated and upregulated in these 3 clones, respectively (Fig. 1B). Downregulation of E-cadherin mRNA and upregulation of N-cadherin mRNA were also observed in cisplatin^r^/LoVo #10, #19, and #54 cells (Fig. 1C). Furthermore, Slug, an EMT-inducing transcription factor (EMT-TF), was upregulated at both the mRNA and protein levels in cisplatin^r^/LoVo #19 cells (Figs. 1B and 1C). Other EMT-TFs, including ZEB2 and Twist, were also upregulated in cisplatin^r^/LoVo #10, #19, and #54 cells at RNA levels (Fig. 1C). On the other hand, the expression of other mesenchymal marker vimentin was slightly increased in cisplatin^r^/LoVo #10, #19, and #54 cells (Fig. 1B). These results suggested that cisplatin^r^/LoVo cells obtained mesenchymal-like features, but their transitions were incomplete. Next, we checked the sensitivities of cisplatin^r^/LoVo #10, #19, and #54 cells against cisplatin. Cisplatin at 30 μM increased the sub-G1 population in LoVo cells (49.3%) but did not increase in cisplatin^r^/LoVo #10, #19, and #54 cells (Fig. 1D). On the other hand, cisplatin induced cell cycle arrest at G2/M phase in both parental LoVo cells and cisplatin^r^/LoVo cells (Fig. 1D and Table 2). Moreover, compared to LoVo cells, cisplatin^r^/LoVo #10, #19, and #54 cells exhibited approximately two-fold higher proliferation rate of the cells in 2D *vs*. 3D cultures (Suppl. Fig. 1).

**Table 2:** Cell cycle and sub-G1 distributions shown in Fig. 1D

		Cisplatin (μM)											
		0				10				30			
		Sub-G1	G1	S	G2/M	Sub-G1	G1	S	G2/M	Sub-G1	G1	S	G2/M
LoVo		0.6%	64.0%	18.8%	16.8%	3.4%	11.2%	17.9%	67.0%	49.3%	33.2%	8.5%	9.2%
cisplatin^r^/LoVo	#10	0.5%	67.7%	15.2%	16.8%	3.9%	15.7%	21.4%	59.5%	6.8%	39.9%	26.3%	27.4%
	#19	0.4%	65.8%	16.3%	17.6%	5.3%	15.0%	26.8%	53.5%	13.4%	43.9%	19.8%	23.2%
	#54	1.3%	73.4%	11.7%	14.0%	1.9%	25.7%	13.9%	58.9%	3.1%	49.8%	22.3%	25.2%

### TGF-β inhibitors partially reverse mesenchymal-like phenotypes of cisplatin^r^/LoVo cells

**Figure 2:**
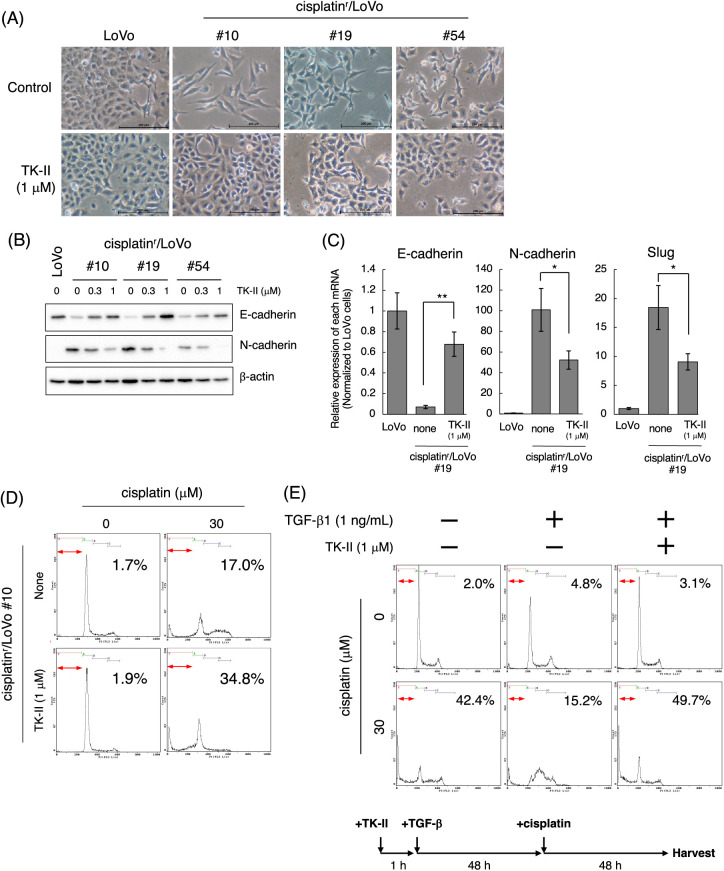
TβRI kinase inhibitors partially reverse the mesenchymal-like phenotype of cisplatin^r^/LoVo cells to epithelial-like phenotype. (A) Morphological changes of LoVo and cisplatin^r^/LoVo cells after TK-II treatment. LoVo or cisplatin^r^/LoVo cells (#10, #19, and #54) were treated with 1 μM TK-II for 72 h, and their morphologies were observed under a phase-contrast microscope. Images are representative of two independent experiments. Scale bar, 200 μm. (B) The effect of TK-II on E-cadherin and N-cadherin expression in cisplatin^r^/LoVo cells. Cisplatin^r^/LoVo cells were treated with 0.3 or 1 μM TK-II for 72 h. Expression of E-cadherin and N-cadherin was evaluated by western blotting. Images are representative of two independent experiments. (C) The effect of TK-II on the expression of E-cadherin, N-cadherin, and Slug mRNAs in cisplatin^r^/LoVo #19 cells. Cisplatin^r^/LoVo #19 cells were treated with 1 μM TK-II for 72 h. Then, the amount of E-cadherin, N-cadherin, and Slug mRNAs were evaluated with qPCR. The presented data are normalized to LoVo cells. All data are representative of three independent experiments and presented as mean SD ± (n = 3). *p < 0.05, **p < 0.01 (two-tailed Student’s test). (D) Effect of TK-II on cisplatin-induced apoptosis of cisplatin^r^/LoVo cells. Cisplatin^r^/LoVo#10 cells were pre-treated with 1 μM TK-II for 72 h and then were treated with 30 μM cisplatin for 48 h. Cells were fixed with 70% ethanol, stained with PI, and analyzed using flow cytometry. Percentages of the subG1 population (areas between red line) in cells are shown. Images are representative of two independent experiments. (E) TGF-β1-pre-treated LoVo cells are resistance to cisplatin-induced apoptosis. TK-II was pre-treated 1 h before the treatment of cells with 1 ng/mL TGF-β1 for 48 h. Then, the cells were further treated with 0 or 30 μM cisplatin for 48 h. Then, the cells were harvested and fixed with 70% ethanol, stained with PI, and analyzed using flow cytometry. The number indicates the subG1 population (areas between red line). Images are representative of two independent experiments. The schematic illustration shows a diagram of the treatment protocol.

To address the molecular mechanism by which continuous exposure to cisplatin alters the epithelial-like phenotype of LoVo cells to a mesenchymal-like phenotype, we searched for compounds that can reverse the mesenchymal-like phenotype of cisplatin^r^/LoVo cells to the epithelial-like phenotype using SCAD inhibitor kits (a gift from the Screening Committee of Anticancer Drugs). Among them, TβRI kinase inhibitors were found to partially reverse their phenotype. As presented in Fig. 2A, the morphologies of cisplatin^r^/LoVo #10, #19, and #54 cells partially changed to a tight cell-to-cell contact with 1 μM TK-II [17] at which TGF-β-induced Smad3 phosphorylation, E-cadherin downregulation, and N-cadherin upregulation were completely suppressed in LoVo cells (Suppl. Fig. 2). TK-II dose-dependently increased E-cadherin expression and decreased N-cadherin expression in cisplatin^r^/LoVo cells (#10, #19, #54) (Fig. 2B). Moreover, 1 μM TK-II increased E-cadherin mRNA in cisplatin^r^/LoVo #10, #19, and #54 cells, whereas it decreased N-cadherin mRNA (Fig. 2C and Suppl. Fig. 3A). 1 μM TK-II also decreased the mRNA and protein expression of Slug in cisplatin^r^/LoVo #19 cells (Fig. 2C and Suppl. Fig. 3B). Curiously, it only marginally affected ZEB2 and Twist mRNAs (Suppl. Fig. 3C). Another TβRI kinase inhibitor, SB431452 [18], also slightly increased E-cadherin expression and decreased N-cadherin expression of cisplatin^r^/LoVo #19 cells. However, the inhibitory effects of SB431542 at 1 μM seemed to be weaker than those of TK-II at 1 μM (Suppl. Fig. 4). These might be due to the different sensitivities between SB431542 and TK-II against TGF-β signaling because the inhibitory effects of SB431542 on LoVo cells were about 10-fold weaker than those of TK-II (Suppl. Fig. 2B). These results suggested that TβRI kinase inhibitors partially reversed cisplatin^r^/LoVo cells from the mesenchymal-like phenotype to the epithelial-like phenotype. We next examined if the TβRI kinase inhibitor improves the sensitivity of cisplatin^r^/LoVo cells against cisplatin. As presented in Fig. 2D, while 30 μM cisplatin induced apoptosis in approximately 17% of cisplatin^r^/LoVo #10 cells, whereas cisplatin-induced apoptosis was approximately 35% of them when they were pre-treated with 1 μM TK-II. These results suggest that TGF-β signaling is involved not only in cisplatin-induced the mesenchymal-like phenotypic change but also in resistance to cisplatin-induced apoptosis. To confirm our hypothesis, we further examined if TGF-β-stimulated LoVo cells acquire cisplatin resistance. Upon the treatment of LoVo cells with 1 ng/mL TGF-β1 for 48 h, expression of E-cadherin and N-cadherin were increased and decreased, respectively (Suppl. Fig. 2B). Therefore, the obtained TGF-β1-induced mesenchymal-like LoVo cells were treated with 30 μM cisplatin. As seen in Fig. 2E, 30 μM cisplatin induced apoptosis in approximately 42.4% of LoVo cells, whereas it was unable to induce apoptosis in mesenchymal-like LoVo cells induced by TGF-β1 (15.2%). Apoptotic resistance against cisplatin in mesenchymal-like LoVo cells was canceled by pre-treatment with 1 μM TK-II (Fig. 2E). Thus, mesenchymal-like LoVo cells induced by TGF-β1 might become resistant to cisplatin-induced apoptosis. Taken together, these results suggest that TGF-β signal pathway might play an important role in the acquisition of cisplatin resistance.

### Cisplatin induces the mesenchymal-like phenotypic change through the activation of TGF-β signal

**Figure 3:**
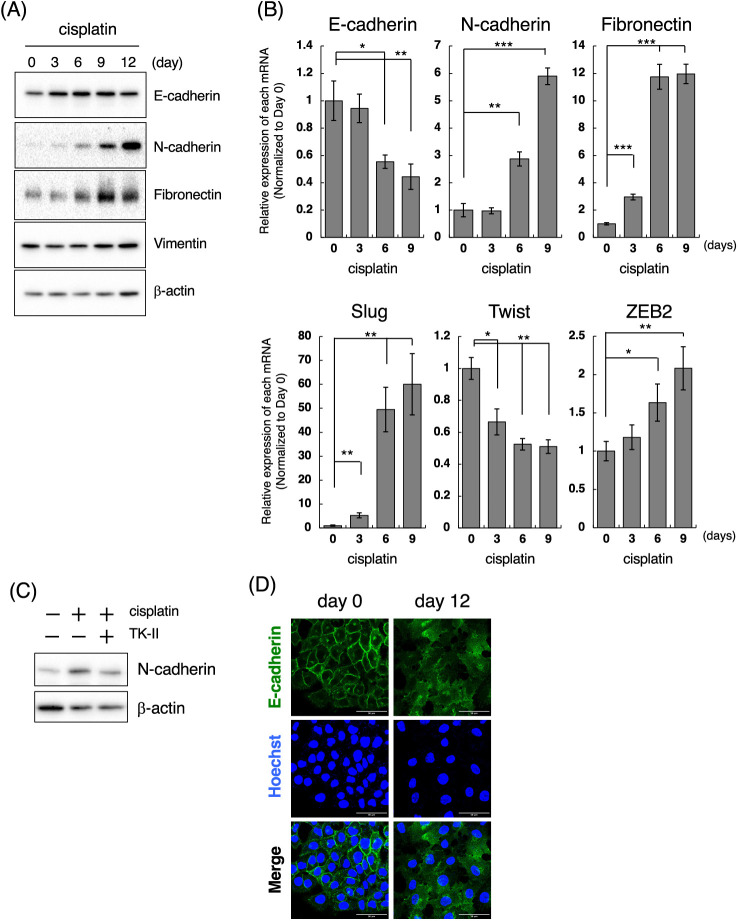
Cisplatin induces the mesenchymal-like phenotypic change. (A) Changes in the protein expression of EMT markers after the treatment of LoVo cells with cisplatin. LoVo cells were treated with 1 μM cisplatin for the indicated periods (0, 3, 6, 9, and 12 days). The protein expression of E-cadherin, N-cadherin, Fibronectin, Vimentin, and β-actin was detected by western blotting. Images are representative of two independent experiments. (B) The effect of cisplatin on mRNA expression of EMT markers in LoVo cells. LoVo cells were treated with 1 μM cisplatin for the indicated periods (0, 3, 6, and 9 days). The levels of E-cadherin, N-cadherin, Fibronectin, Slug, Twist, and ZEB2 mRNAs were evaluated using qPCR. The presented data are normalized to Day 0. All data are representative of three independent experiments and presented as mean SD ± (n = 3). *p < 0.05, **p < 0.01, ***p < 0.001 (two-tailed Student’s test). (C) The TβRI kinase inhibitor suppressed cisplatin-induced N-cadherin upregulation in LoVo cells. LoVo cells were treated with 1 μM cisplatin and/or 1 μM TK-II. Six days later, the cells were collected and subjected to western blot analysis to detect N-cadherin and β-actin expression. Images are representative of two independent experiments. (D) Cisplatin affects the subcellular localization of E-cadherin. LoVo cells were treated with 1 μM cisplatin. Twelve days later, the cells were fixed and stained with an anti-E-cadherin antibody. Subcellular localization of E-cadherin was observed using confocal laser scanning microscope. Images are representative of two independent experiments. Scale bar, 50 μm.

According to the above results, we next examined when cisplatin induces mesenchymal-like phenotypic change in LoVo cells. For those purposes, we performed time-course experiments after the treatment of cells with cisplatin at low concentrations. When 1 μM cisplatin was exposed to LoVo cells, expression of N-cadherin and Fibronectin were increased in time-course dependent manner (Fig. 3A). Upregulation of N-cadherin and Fibronectin mRNAs was also observed after treatment of LoVo cells with cisplatin (Fig. 3B). Interestingly, the cisplatin-induced upregulation of N-cadherin was suppressed by TK-II (Fig. 3C), suggesting that N-cadherin upregulation by the exposure of cisplatin to LoVo cells is possibly due to the activation of TGF-β signal. On the other hand, the expression of Vimentin was not altered (Fig. 3A). Moreover, the protein expression of E-cadherin was not affected by cisplatin treatment (Fig. 3A), although E-cadherin mRNA was downregulated by about 50% after 6 days treatment of LoVo cells with cisplatin (Fig. 3B).

E-cadherin, a cell membrane-associated protein involved in cell-cell adhesion, plays a key role in inducing cell polarity and organizing the epithelium. Because the loss of E-cadherin along cell-cell junction is considered as a hallmark of EMT [19,20], we examined the subcellular localization of E-cadherin in cisplatin-treated LoVo cells. While E-cadherin was localized along cell-cell junction under normal conditions, E-cadherin expression along cell-cell junction could not be observed at 12 days after treatment of LoVo cells with cisplatin (Fig. 3D). These results suggest that cisplatin causes loss of epithelial cell polarity. We also investigated the effects of cisplatin on the expression of EMT-TFs. As presented in Fig. 3B, Slug mRNA expression was increased by approximately 5-fold or 50-fold at 3 and 6 days after treatment of LoVo cells with cisplatin, respectively. ZEB2 mRNA expression was also increased by cisplatin treatment although the inducibility of ZEB2 mRNA by cisplatin was weaker than that of Slug mRNA by cisplatin. In contrast, the Twist mRNA expression was decreased after cisplatin treatment (Fig. 3B). Taken together, these results propose that cisplatin might induce the mesenchymal-like phenotypic change in LoVo cells within one week.

### Cisplatin-induced mesenchymal-like phenotypic change occurs in cell lines highly responsive to TGF-β

**Figure 4:**
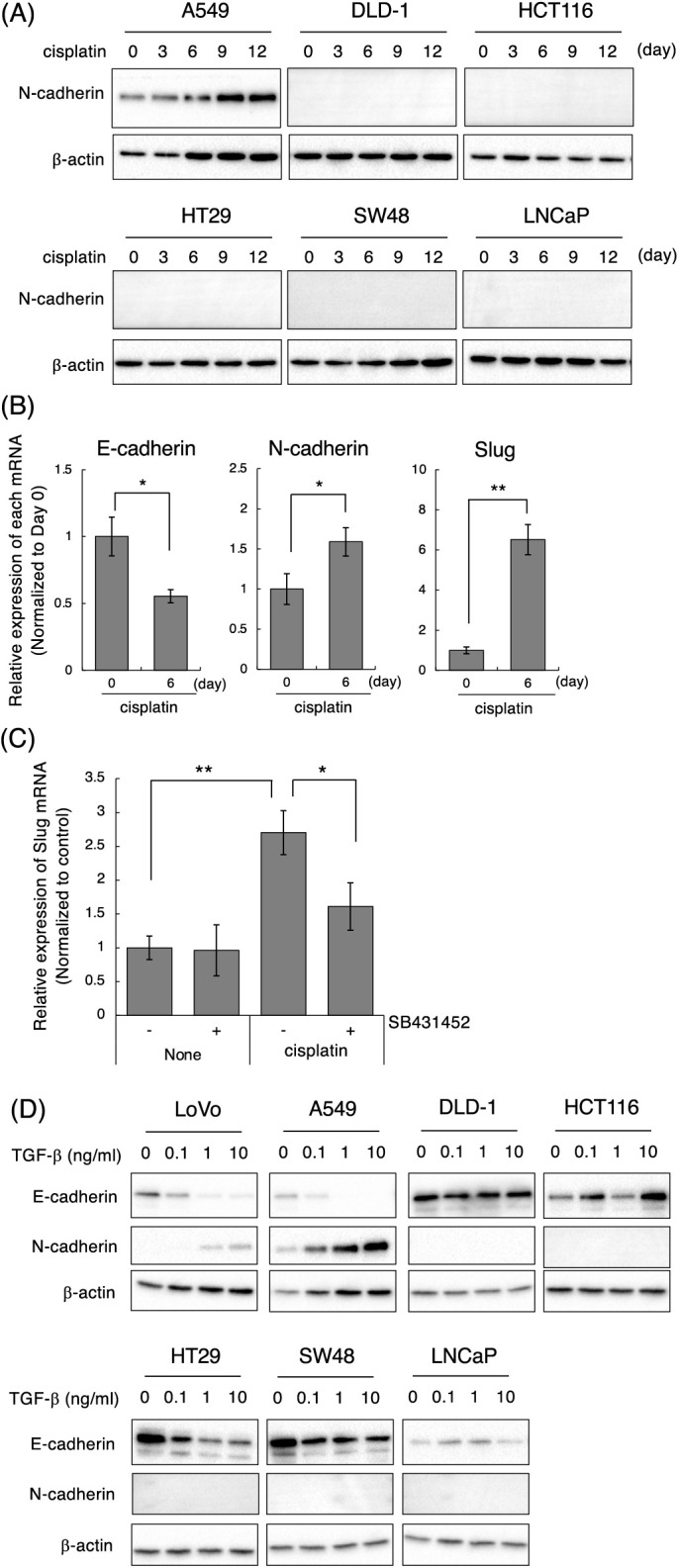
Effect of cisplatin on various types of cancer cell lines. (A) Cisplatin enhanced N-cadherin expression in A549 but not in DLD-1, HCT116, HT29, SW48, and LNCaP cells. Cells were treated with 1 μM cisplatin for the indicated periods (0, 3, 6, 9, and 12 days). The protein expression of N-cadherin and β-actin was detected by western blotting. Images are representative of two independent experiments. (B) Cisplatin decreased E-cadherin mRNA, and increased N-cadherin and Slug mRNAs in A549 cells. A549 cells were treated with 1 μM cisplatin for the indicated periods. The amount of E-cadherin, N-cadherin, and Slug mRNAs were evaluated using qPCR. The presented data are normalized to Day 0. All data are representative of three independent experiments and presented as mean SD ± (n = 3). *p < 0.05 and **p < 0.01 (two-tailed Student’s test). (C) The TβRI kinase inhibitor, SB431452, partially suppressed cisplatin-induced Slug upregulation in A549 cells. A549 cells were treated with 10 μM cisplatin and/or 1 μM SB431452. Twenty-four hours later, the cells were collected and subjected to qPCR analysis to detect Slug mRNA. The presented data are normalized to control cells. All data are representative of three independent experiments and presented as mean SD ± (n = 3). *p < 0.05 and **p < 0.01 (two-tailed Student’s test). (D) TGF-β1 induces and decreases N-cadherin and E-cadherin in LoVo and A549 cells. Cells were stimulated with the indicated concentrations of TGF-β1 (0, 0.1, 1, 10 ng/mL). Two days later, the cells were collected and subjected to western blot analysis to detect E-cadherin, N-cadherin, and β-actin.

We further investigated the cell type specificity of cisplatin-induced the mesenchymal-like phenotypic change. To conduct this, we selected epithelial cell lines by evaluating the morphology and the expression of E-cadherin and N-cadherin in 16 cancer cell lines including lung, colon, prostate, and breast cancers. As a result, NSCLC adenocarcinoma A549, colorectal adenocarcinoma DLD-1, HCT116, HT29, and SW48 cells as well as prostate adenocarcinoma LNCaP cells showed epithelial-like morphologies. Furthermore, the expression of E-cadherin was high and the expression of N-cadherin was low or undetectable in these cell lines (data not shown). Therefore, we tested whether cisplatin induces the mesenchymal-like phenotypic change in these cell lines by evaluating the protein expression of N-cadherin, which was upregulated by cisplatin at both the mRNA and protein levels in LoVo cells. Next, we determined the concentrations of cisplatin. As shown in Suppl. Fig. 5A, the sensitivities of cisplatin against A549, DLD-1, HCT116, HT29, SW48, and LNCaP cells were similar to that of LoVo cells. Therefore, we treated with 1, 3, 10 μM cisplatin for 12 days and checked whether cisplatin-treated cancer cells could be survived after 12 days of treatment. As a result, because 1 μM cisplatin induced the morphological changes and growth arrest without inducing cell death (data not shown), we determined the concentration of cisplatin as 1 μM. As presented in Fig. 4A, N-cadherin protein expression was increased at 6 or 9 days after the treatment of A549 cells with cisplatin. In A549 cells, cisplatin decreased E-cadherin mRNA, and increased N-cadherin and Slug mRNAs, although it did not affect the expression levels of Twist and ZEB2 mRNAs (Fig. 4B and data not presented). Moreover, cisplatin-induced upregulation of Slug mRNA in A549 cells was partially suppressed by SB431542 (Fig. 4C). These results indicate that cisplatin induces the mesenchymal-like phenotypic change through the activation of TGF-β signaling in A549 cells like LoVo cells. In contrast, cisplatin did not induce N-cadherin expression in DLD-1, HCT116, HT29, SW48, and LNCaP cells, although the sensitivities of these cell lines to cisplatin were comparable (Suppl. Fig. 5A). To investigate the correlation between TGF-β and cisplatin-induced the mesenchymal-like phenotypic change in these cell lines, we evaluated TGF-β-induced E-cadherin downregulation and N-cadherin upregulation. As shown in Fig. 4D, E-cadherin downregulation and N-cadherin upregulation were not observed in DLD-1, HCT116, SW48, and LNCaP cells even though cells were stimulated with 10 ng/mL TGF-β1 unlike in LoVo and A549 cells. On the other hand, while E-cadherin downregulation was observed in HT29 cells, N-cadherin upregulation was not. When we examined if these cells could respond to TGF-β1, Smad3 phosphorylation was undetectable in DLD-1 and LNCaP cells and slightly below the limit of detection in HCT116, HT29, and SW48 cells although Smad3 phosphorylation was detected in LoVo and A549 cells (Supplementary Fig. 5B). Thus, it was suggested that cisplatin-induced the mesenchymal-like phenotypic change was observed in the cells highly responsive to TGF-β.

### Cisplatin enhances the secretion of TGF-β1 into culture media

**Figure 5:**
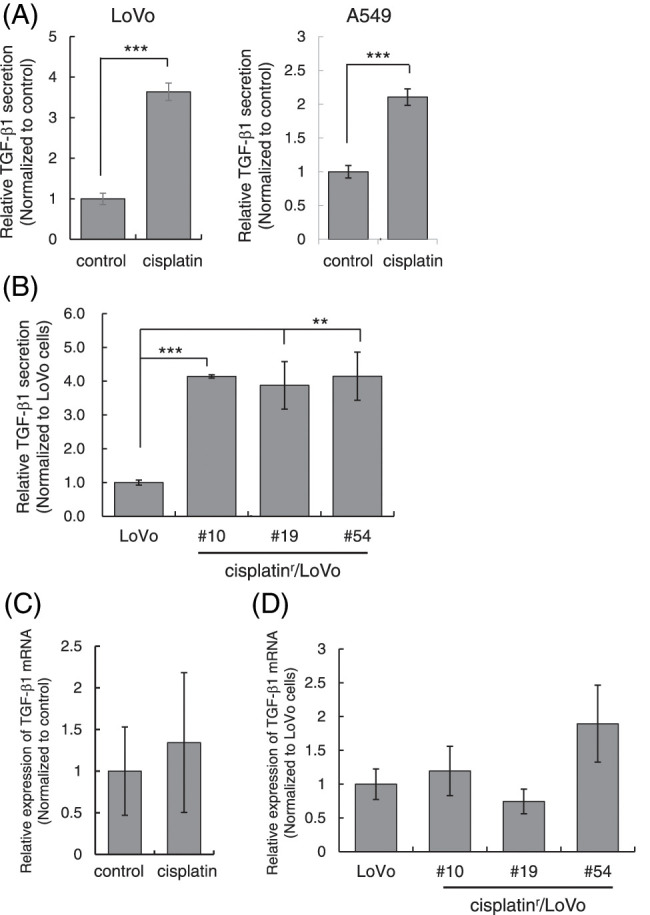
Cisplatin enhances the secretion of TGF-β1. (A) Cisplatin increased the amount of TGF-β1 in the culture media from LoVo and A549 cells. LoVo and A549 cells were treated with 1 μM cisplatin for 72 h. The culture media from cells were collected and subjected to ELISA to measure the amount of TGF-β1. The amounts of TGF-β1 from LoVo and A549 cells without cisplatin were used to calculate relative values. All data are presented as mean SD ± (n = 3). ***p < 0.001 (two-tailed Student’s test). (B) The amount of TGF-β1 in the culture media from cisplatin^r^/LoVo cells were increased. The culture media from LoVo or cisplatin^r^/LoVo (#10, #19, and #54) cells were collected and subjected to ELISA to measure the amount of TGF-β1. The amount of TGF-β1 from LoVo cells without cisplatin was used to calculate relative values. All data are presented as mean SD ± (n = 3). **p < 0.01 and ***p < 0.001 (two-tailed Student’s test). (C) The effect of cisplatin on the expression of TGF-β1 mRNA in LoVo cells. LoVo cells were treated with 1 μM cisplatin. Three days later, the total RNA was extracted and subjected to qPCR analysis to measure the amount of TGF-β1 mRNA. The presented data are normalized to control cells. All data are representative of three independent experiments. (D) The comparison of the expression levels of TGF-β1 mRNA in LoVo and cisplatin^r^/LoVo cells. Total RNA from exponentially growing LoVo or cisplatin^r^/LoVo cells (#10, #19, and #54) was extracted and subjected to qPCR analysis to measure the amount of TGF-β1 mRNA. The presented data are normalized to LoVo cells. All data are representative of three independent experiments.

Previously, cisplatin was reported to stimulate the secretion of TGF-β1 in MDA-MB-231 spheroids [21]. Therefore, we examined if cisplatin enhances TGF-β secretion from cells to activate TGF-β signaling. We measured the amount of TGF-β1 in the culture media after cisplatin treatment by ELISA and normalized using the number of live cells attached to the plate at the time of harvest. As shown in Fig. 5A, the amounts of TGF-β1 in the culture media from both LoVo and A549 cells were significantly increased when cells were stimulated with 1 μM cisplatin for 72 h. Furthermore, we measured the amounts of TGF-β1 in the culture media from cisplatin^r^/LoVo cells. As a result, the amounts of TGF-β1 in the culture media from cisplatin^r^/LoVo cells (#10, #19, and #54) was about 4-fold higher than that of LoVo cells (Fig. 5B). These results indicate that the mesenchymal-like phenotype of cisplatin^r^/LoVo cell might be accompanied by the activation of TGF-β signal. Curiously, the expression of TGF-β1 mRNA was not increased by cisplatin in LoVo cells (Fig. 5C). Furthermore, the mRNA expression of TGF-β1 in cisplatin^r^/LoVo cells was not statistically higher than that in LoVo cells (Fig. 5D). These results suggest that cisplatin might enhance the secretion of TGF-β1 into the culture media without the influence of the transcription of the TGF-β1 gene.

## Discussion

In this study, we show the following observations: (1) The TβRI kinase inhibitors partially change cisplatin^r^/LoVo cells from mesenchymal-like phenotypes to epithelial-like phenotype; (2) the secretion of TGF-β1 is enhanced in both cisplatin^r^/LoVo cells and cisplatin-treated LoVo cells; (3) cisplatin induces the mesenchymal-like phenotypic change in cell lines highly responsive to TGF-β1. According to these observations, we suggest that TGF-β signal plays a critical role in cisplatin-induced the mesenchymal-like phenotypic change. Because TβRI mRNA and TβRII mRNA were slightly increased in cisplatin^r^/LoVo #19 cells compared to LoVo cells (data not shown), it remains the possibility that the enhancement of TGF-β1 secretion and the increased expression of TβRI and TβRII synergistically activate the TGF-β signaling.

Although cisplatin induced the mesenchymal-like phenotypic change in LoVo and A549 cells, it did not induce it in the other five cell lines (DLD-1, HCT116, HT29, SW48, and LNCaP) examined. Among them, DLD-1 was reported to carry TβRI*6A/*9A genotype. TβRI*6A is one of the first identified susceptibility alleles. The variant TβRI*6A encodes the deletion of 3 alanines within a 9-alanine repeat (*9A) at the 3'-end of the exon 1 coding sequence. Consequently, TβRI*6A is less sensitive to TGF-β than the wild-type TβRI [22,23]. Furthermore, HCT116 cell line was reported to carry a mutation in TβRII. Thus, TGF-β failed to promote phosphorylation of Smad2 in DLD-1 and HCT116 cells [24]. Moreover, the expression of TβRI was undetected in LNCaP cells [25]. In addition, HT-29 cells are known to be Smad4 negative cells. HT29 cells have a nonsense mutation in the exon 4 of the Smad4 gene, in which its MH2 domain is included. Thus, this truncated form of Smad4 cannot make a complex with R-Smads (Smad2 and/or Smad4) when cells are stimulated with TGF-β [26,27]. SW48 cells was shown to be insensitive to TGF-β [28]. Therefore, we were unable to detect TGF-β1-induced Smad3 phosphorylation, E-cadherin downregulation nor N-cadherin upregulation in DLD-1, HCT116, HT29, SW48, and LNCaP cells. These results support the importance of TGF-β signaling in cisplatin-induced the mesenchymal-like phenotypic change.

EGFR tyrosine kinase inhibitors (EGFR-TKIs), such as gefitinib and erlotinib, are clinically available for the treatment of non-small lung cancers (NSCLC). However, chronic exposure of EGFR-mutated NSCLC cells to EGFR-TKIs is known to lead them to acquire EGFR-TKI’s resistance. Soucheray et al. have reported that EGFR-TKIs enhanced the secretion of TGF-β1 into the culture media to activate TGF-β/Smad signaling that is involved in EMT induction and apoptosis resistance [29]. Consistent with their results, we also observed the enhanced secretion of TGF-β1 in LoVo cells treated with cisplatin for acquirement of an anti-cancer resistance. Wang et al. also supported our results because the reduced expression of TGF-β1 in cisplatin-resistant A549 cells altered them from the mesenchymal phenotype to the epithelial phenotype to enhance the sensitivity against cisplatin [30]. Long-term administration of doxorubicin increased the concentration of TGF-β1 in the culture media, which in turn prompted the activation of the TGF-β/Smad4 pathway and the induction of EMT [31]. On the other hand, it is still unclear how anti-cancer drugs enhance the secretion of TGF-β1. TGF-β is synthesized as a latent precursor consisting of a TGF-β homodimer which is non-covalently linked to the latency associated protein, forming a small latent complex (SLC). Latent TGF-β binding protein (LTBP) makes a complex with SLC at the extracellular matrix as a large latent complex (LLC) [32] in which LTBP protects degradation of SLC [33]. TGF-β activation involves the removal of both TGF-β binding proteins and latency-associated proteins, and occurs through proteolytic processes including plasmin [34] and matrix metalloproteinases (MMPs) [35], or non-proteolytically process through conformational changes including integrin and/or mechanical stress [36]. Some anti-cancer drugs, including anthracycline, were reported to induce ECM remodeling by stimulating MMP production and modulating integrin expression [37]. Consistently, doxodubicin, a member of anthracycline could increase N-cadherin expression in LoVo cells, but camptothecin and paclitaxel did not (data not shown). Therefore, it is possible that DNA-damaging agents including cisplatin and doxorubicin change the expression of MMP and/or integrin, which may lead to the enhancement of TGF-β secretion.

In this study, we used the combination of cisplatin with colorectal adenocarcinoma LoVo cells as an *in vitro* model of anti-cancer drug resistance. However, cisplatin is not clinically applicable for colon cancer, although cisplatin is used for a wide range of solid cancers such as ovarian, lung, and head and neck cancer [38]. Therefore, further study is required to investigate whether cisplatin is able to enhance the secretion of TGF-β1, to induce mesenchymal-like phenotypic change, and to acquire cisplatin resistance in cancers where cisplatin is clinically available. Furthermore, it is also necessary what kind of anti-cancer drugs having different modes of action from cisplatin would enhance the secretion of TGF-β1. Taken together with our observation that TβRI kinase inhibitor partially improves the resistance to cisplatin-induced apoptosis in LoVo cells, these experiments would provide fruitful information for the therapeutic application of TβRI kinase inhibitors against anti-cancer drug-resistant tumors. Currently, specific inhibitors of TGF-β signaling including neutralizing antibodies, ligand traps, small-molecule kinase inhibitors, and antisense oligonucleotides have been reported [39]. These inhibitors would be also applicable for anti-cancer drug resistant tumors. TGF-β1 secreted from cancer-associated fibroblasts (CAFs) and tumor-associated macrophages (TAMs) has key roles in cancer progression. Our finding suggests that chemically stressed cancer cells might secrete TGF-β1 for escaping from chemical stresses, which results in their transformation into further malignant cancers.

## Supplementary Materials











## Data Availability

All data supporting the conclusion of this study have been included in this article.

## Supplementary Materials

The supplementary material is available online at https://doi.org/10.32604/or.2023.030190.
