# Supplementary figures and images for: Cisplatin-induced activation of TGF-β signaling contributes to drug resistance
Source: Oncol Res. 2023 Nov 15;32(1):139–50. doi: 10.32604/or.2023.030190 (PMC10767239; doi:10.32604/or.2023.030190)

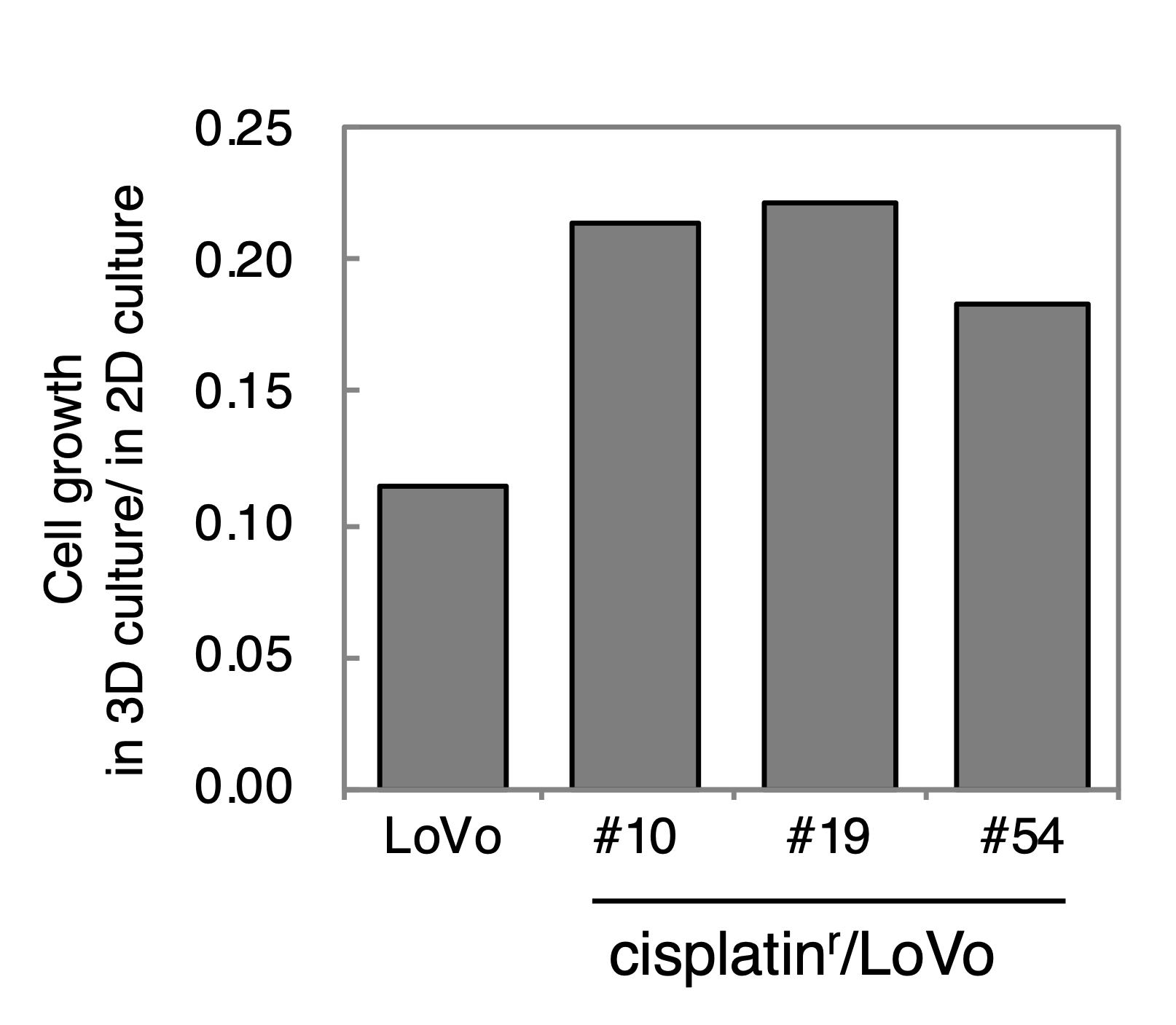

Supplement: Supplementary file 1 [file OncolRes-32-30190-s001.tif]

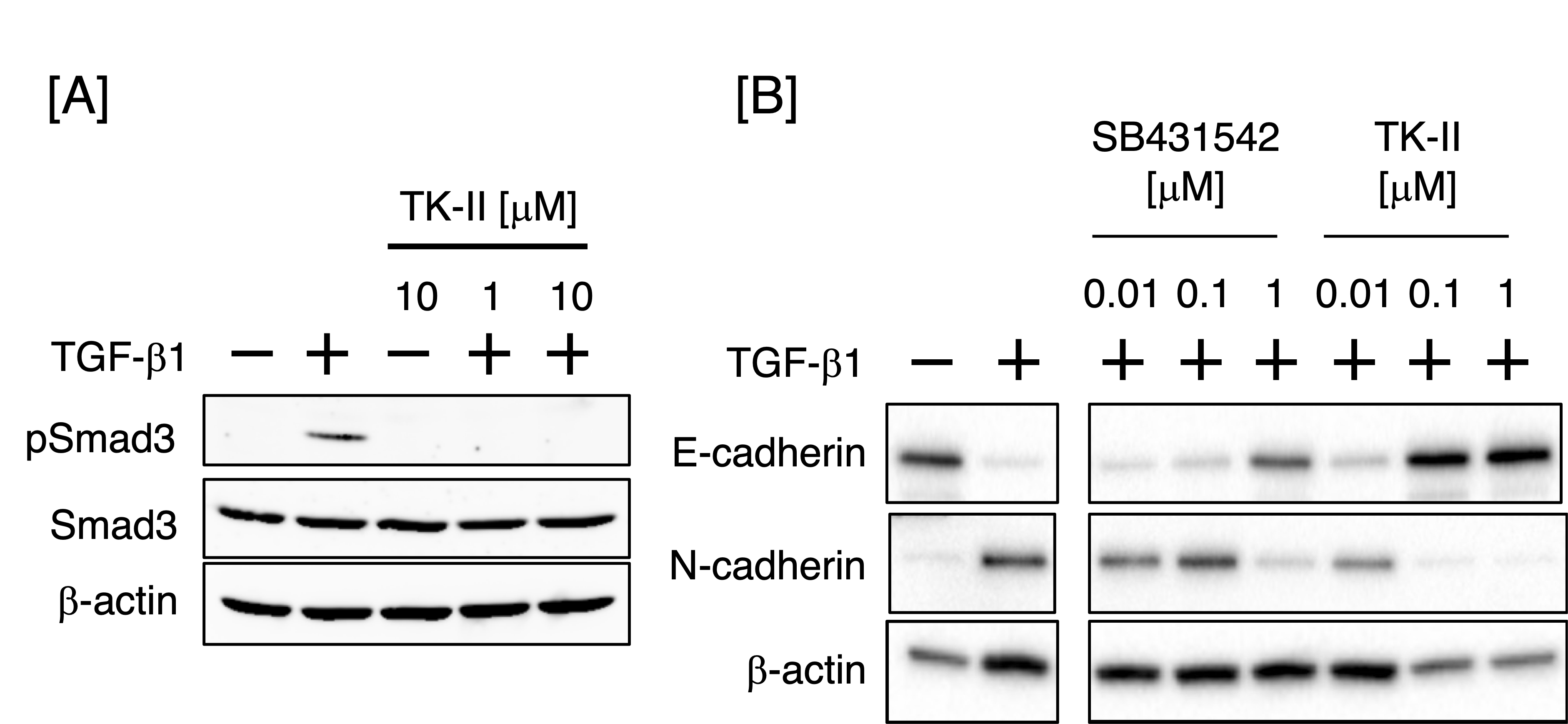

Supplement: Supplementary file 2 [file OncolRes-32-30190-s002.tif]

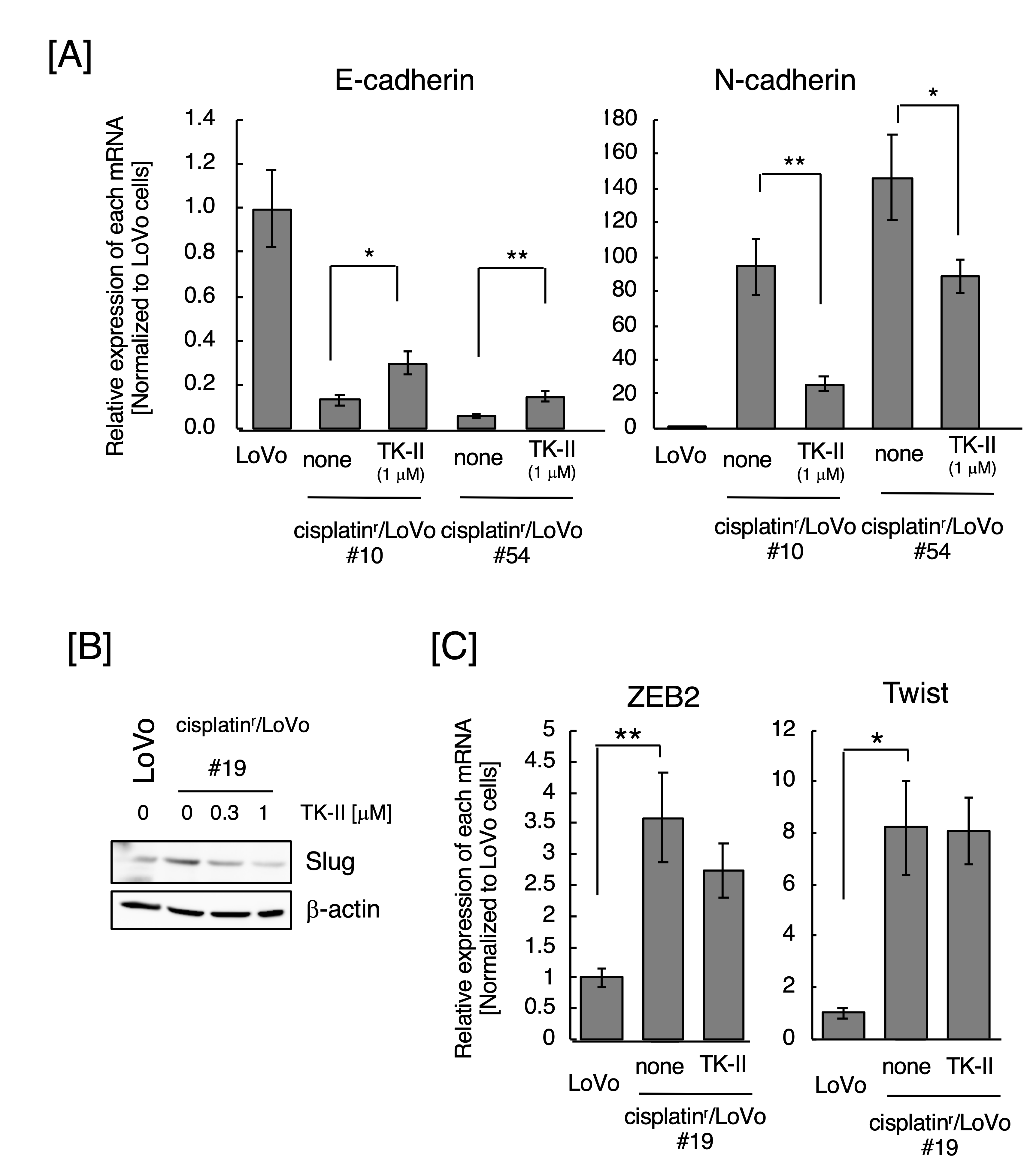

Supplement: Supplementary file 3 [file OncolRes-32-30190-s003.tif]

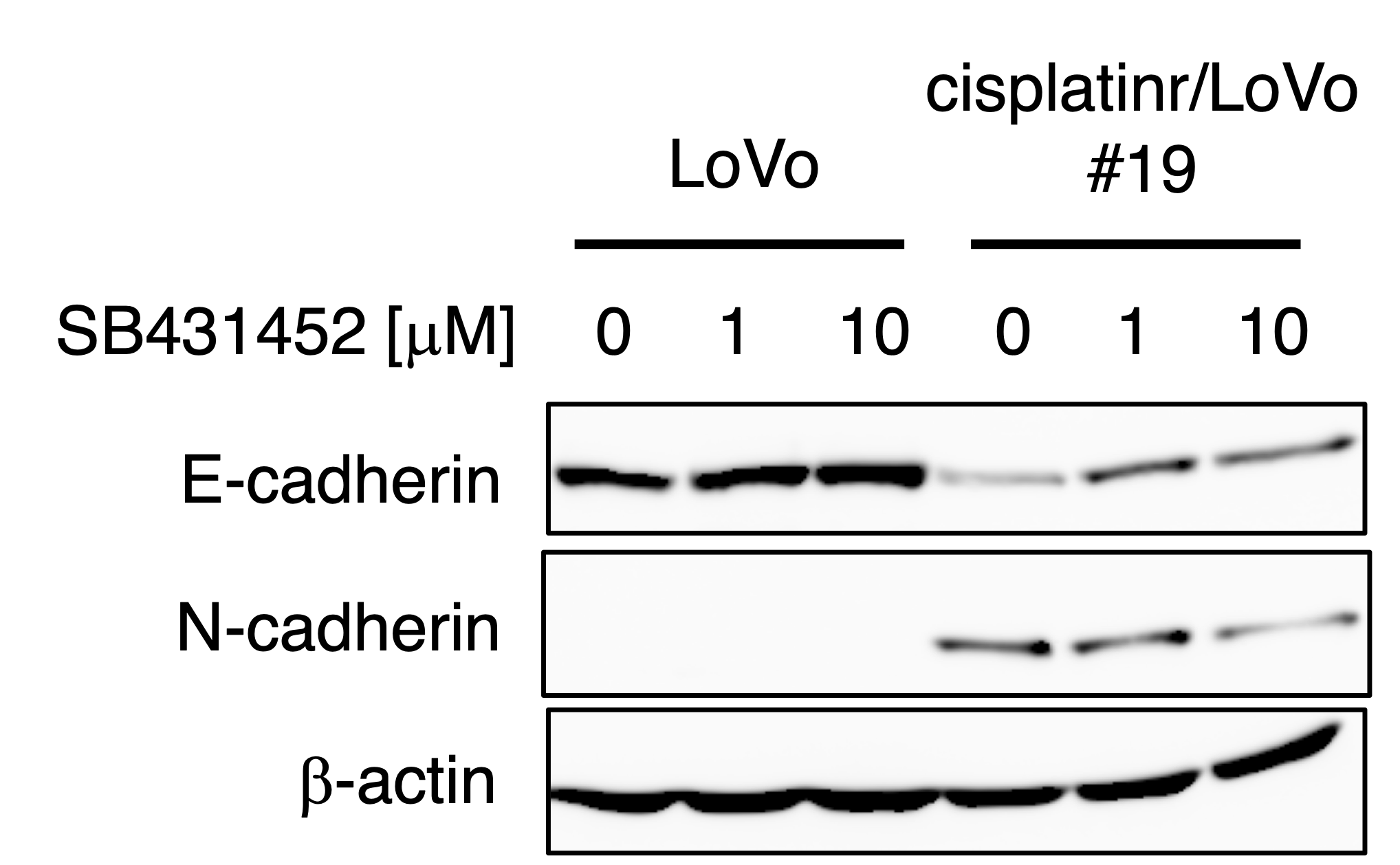

Supplement: Supplementary file 4 [file OncolRes-32-30190-s004.tif]

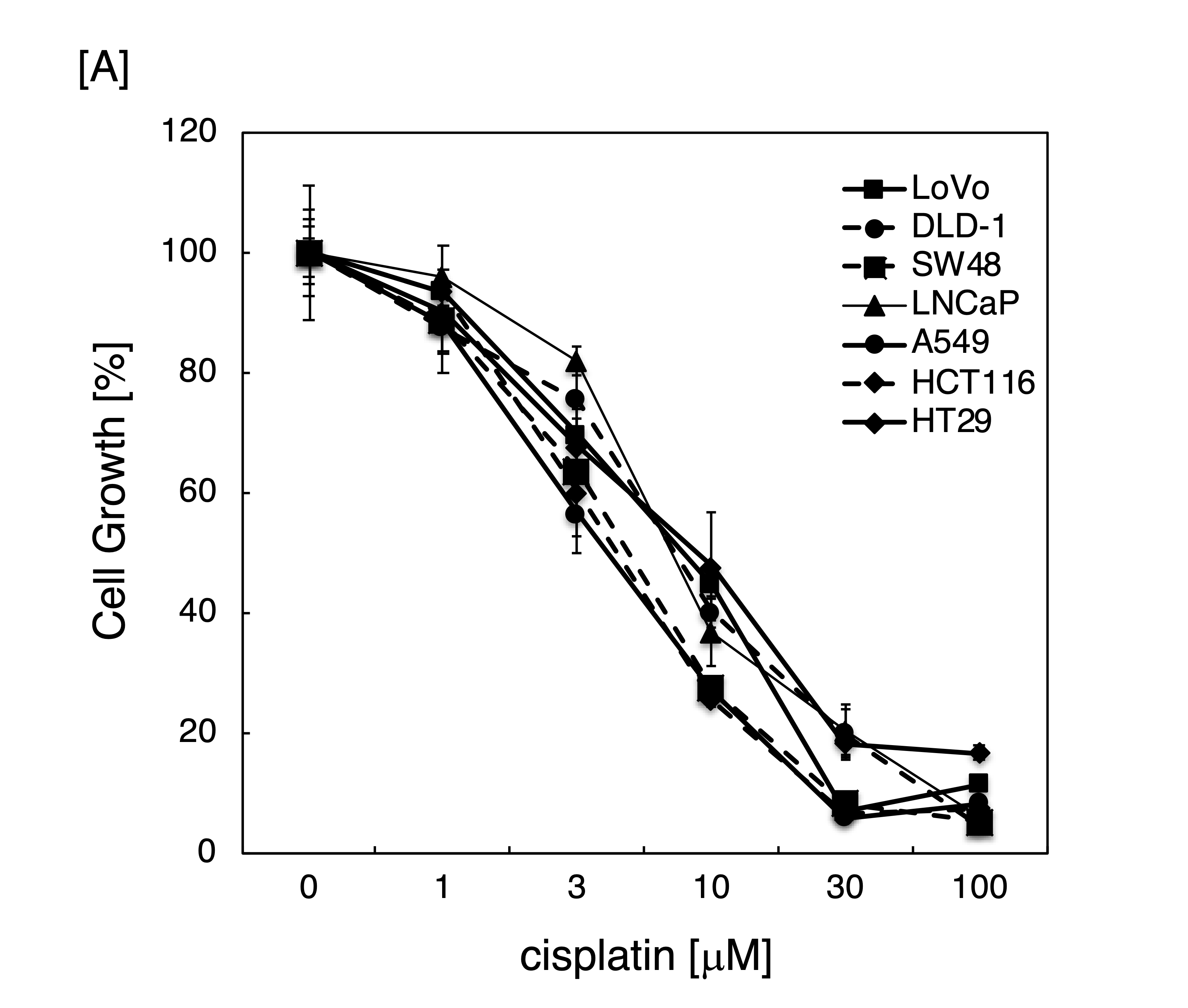

Supplement: Supplementary file 5 [file OncolRes-32-30190-s005a.tif]

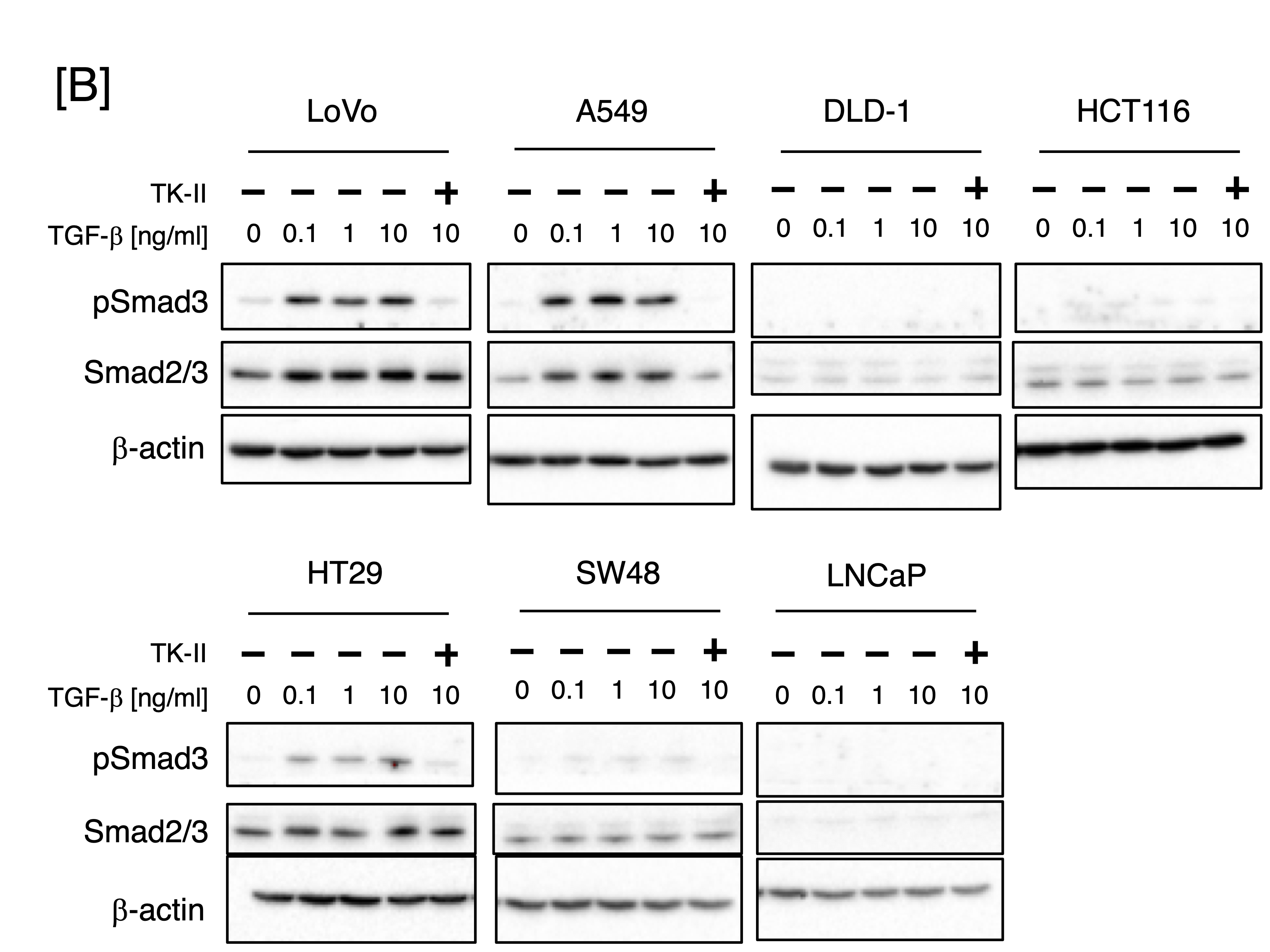

Supplement: Supplementary file 6 [file OncolRes-32-30190-s005b.tif]
